# Congenital medulloblastoma in two brothers with SUFU-mutated Gorlin-Goltz syndrome: Case reports and literature review

**DOI:** 10.3389/fonc.2022.988798

**Published:** 2022-10-12

**Authors:** Yufan Chen, Haibo Zhang, Yang Zhao, Jie Ma

**Affiliations:** Department of Pediatric Neurosurgery, Xinhua Hospital Affiliated to Shanghai Jiao Tong University School of Medicine, Shanghai, China

**Keywords:** congenital medulloblastoma, Gorlin-Goltz syndrome, sufu, stem cell transplantation, infantile

## Abstract

**Background:**

Congenital medulloblastoma is very rare, and many cases involve germline mutations that can lead to inherited syndromes. Here, we first report two brothers with congenital medulloblastoma who were diagnosed with Gorlin-Goltz syndrome caused by SUFU mutation.

**Clinical presentation:**

Medulloblastoma was detected in two brothers at 2 and 3 months of age, with very similar imaging features. Genetic testing revealed that both children and their mother carried SUFU gene germline mutations, and both brothers were diagnosed with Gorlin-Goltz syndrome.

**Conclusion:**

Gorlin-Goltz syndrome-associated congenital medulloblastoma with SUFU germline mutation is very rare. Pathological types mostly involve desmoplastic/nodular or extensive nodularity; chemotherapy is the main treatment, and studies revealing prognostic data are scarce.

## Introduction

Medulloblastoma (MB) is the most common malignant embryonic high-grade brain tumor in children, accounting for approximately 15-20% of pediatric brain tumors. MB occurs in children of all ages, including infants, with a peak age of onset between 5 and 8 years of age (1). In general, congenital brain tumors are intracranial tumors found within 60 days of birth (2). Congenital tumors of the central nervous system (CNS) are classified as “absolutely congenital” (detected at birth) and “probably congenital” (detected within 6 months of age) (3). To date, only 20-30 cases of congenital MB have been reported worldwide, constituting an extremely rare entity (4). Consensus medulloblastoma predisposition genes have not been defined and screening guidelines for genetic counselling and testing for pediatric patients are not available. However, some MB patients with harbor germline mutations that can lead to inherited syndrome, the most common of which is Gorlin-Goltz syndrome, also known as nevoid basal cell carcinoma syndrome (NBCCS), a rare autosomal dominant disease caused by mutations in sonic hedgehog signaling pathway components (5). Overall, there is a lack of uniformity in understanding and treatment of MB-related Gorlin-Goltz syndrome, with mostly case reports in the literature. Moreover, reports on Gorlin-Goltz syndrome-associated congenital MB with SUFU mutation are rare. This study analyzed the clinical data of 2 cases of infantile MB (two brothers) with GS syndrome to improve understanding of the disease.

## Case description

A 3-month-old boy (spontaneous conception; Patient one) with no clinical symptoms visited a local hospital for head MRI examination, which revealed a large mass in the posterior cranial fossa accompanied by obstructive hydrocephalus. The boy was referred to the Pediatric Neurosurgery Department of our hospital. The child showed no signs, such as vomiting or convulsions; no abnormal limb function, growth or development different from peers was present. Patient one underwent MRI examination because a brother (conceived as a result of *in vitro* fertilization because the mother had adenomyosis; Patient two) visited a local hospital at the age of 2 months old owing to vomiting and jaundice; brain MRI examination showed a posterior cranial fossa mass clinically diagnosed as MB based on consultations in multiple hospitals. This local hospital did not have the capacity to carry out operations for children with intracranial tumor, in addition, the parents thought the child was too young for surgery, and did not know enough about the disease at that time, mistakenly learned that the prognosis of MB in young children without further information is not good. In order to avoid the pain caused by the operation, his parents refused surgery and all invasive procedures. Patient two died after 6 months of conservative treatment (severe hydrocephalus, intracranial hypertension).

**Figure 1:**
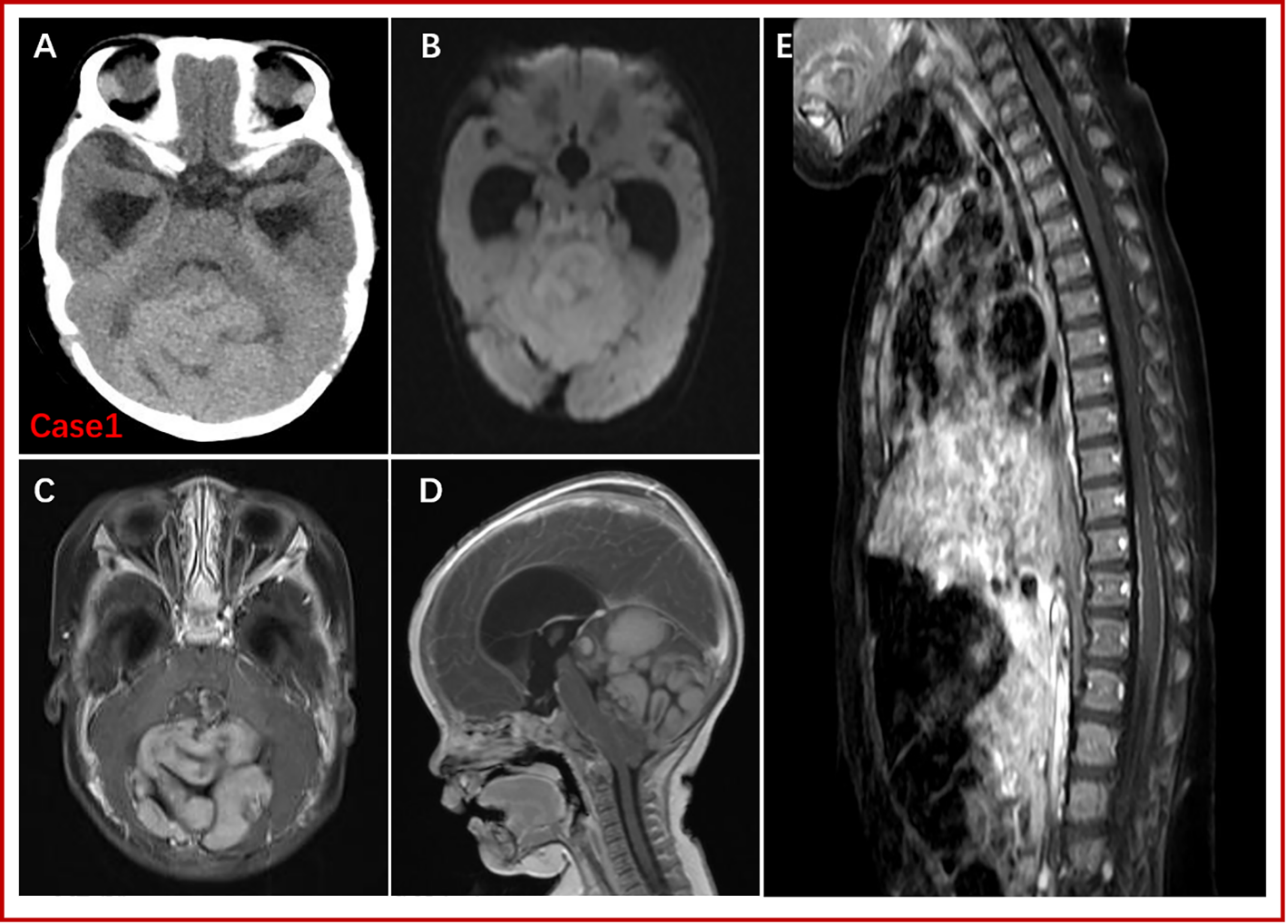
CT and MRI findings for Patient one. A large abnormal signal mass (5.4 cm x 5.7 cm x 7.0 cm) was detected above the cerebellum in the posterior fossa, with poorly defined borders. CT showed a slightly high-density mass shadow. T1WI revealed isointense or low signal, T2-FLAR isointense or slightly high signal, and DWI high signal. Enhanced scanning showed high heterogeneity (multiple nodules). There was no obvious abnormality regarding the spinal cord or morphological signals; the surface of the spinal cord was line-like, with small, strong nodular signals by enhanced scanning. **(A)**: Preoperative, noncontrast CT. **(B)**: DWI. **(C)** and **(D)**: Gd-enhanced T1W images. **(E)**: Gd-enhanced MRI of the spinal cord.

After admission, physical examination of Patient one showed an enlarged head circumference (47 cm), enlarged fontanelle (5*4 cm), and mild sunset eye signs. Head CT revealed a large and slightly high-density mass above the cerebellum in the posterior fossa, approximately 6*6.7 cm in size, with unclear borders, uneven density, and an enlarged ventricle. MRI examination of the head showed a large abnormal signal mass in the posterior cranial fossa, with iso- or hypointensity on T1WI, iso- and slight hyperintensity on T2-FLAIR, and hyperintensity on DWI, with a size of approximately 5.4 cm×5.7 cm×7.0 cm. There were multiple strong nodular contrast-enhanced signals, obvious compression of the brainstem, compression and narrowing of the fourth ventricle, and obvious enlargement of the supratentorial ventricle (**Figure 1**). Enhanced MRI scan of the whole spinal cord indicated no obvious abnormality in the morphology or signal of the spinal cord. Patient one underwent lumbar puncture, no tumor cells were found in CSF examination. According to Chang staging system, Patient one was M0. By comparing with the head MRI of Patient two (**Supplementary Figure 1**), the tumor morphological characteristics of the two boys were very similar.

After all preoperative examinations were completed, contraindications for surgery were excluded, and Ommaya reservoir insertion and external ventricular drainage in the left lateral ventricle were performed. After 3 days, tumor resection of the posterior fossa was carried out. There was no new neurological dysfunction after the operation, and satisfactory physical recovery was achieved. Postoperative head CT and enhanced MRI re-examination indicated satisfactory tumor resection (gross total resection) (**Supplementary Figure 2**).

**Figure 2:**
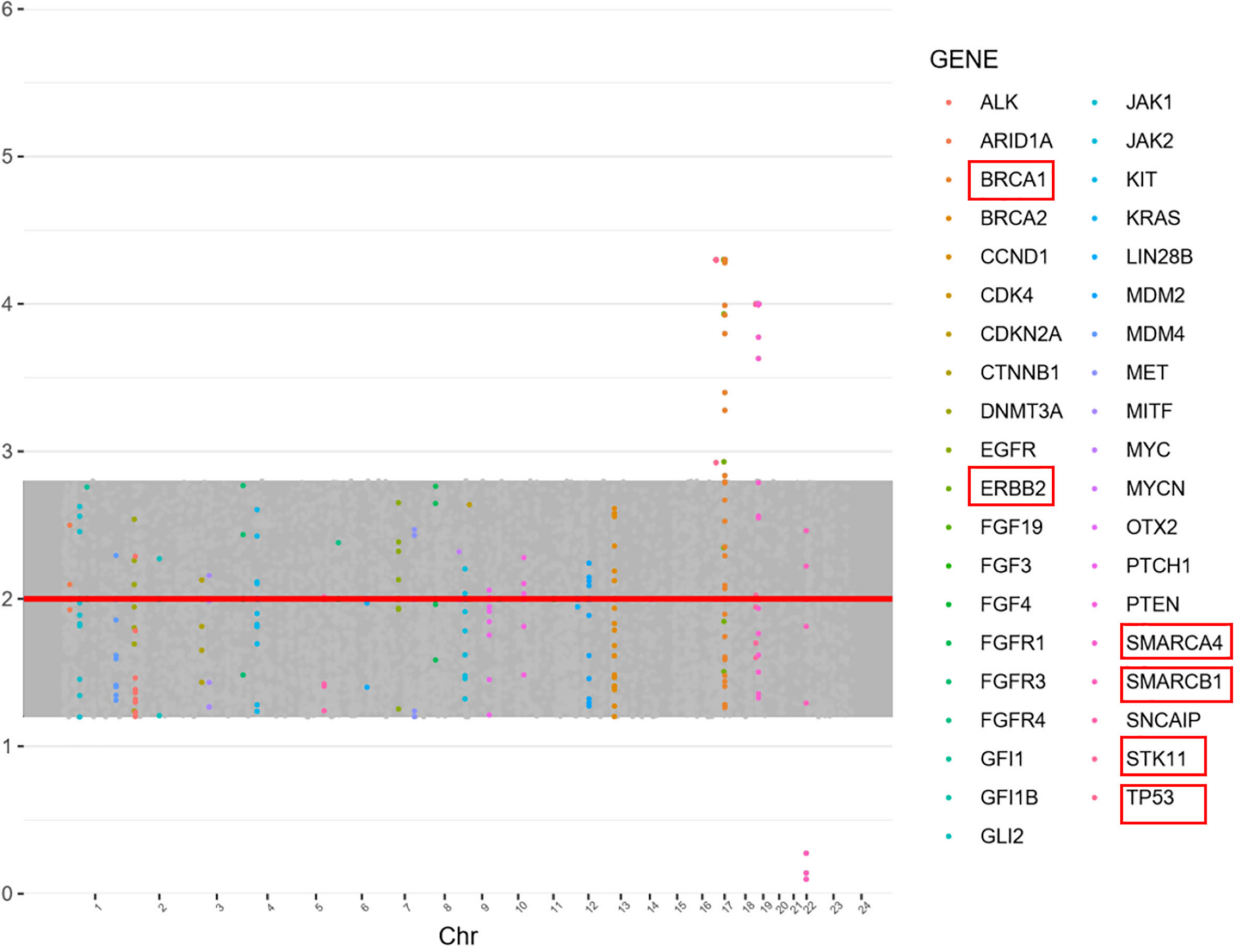
Gene copy number variation (CNV) in Patient one. Copy number was evaluated as the normal copy number: 2, increased 2> and decreasing copy number <2. BRCA1 (3.3,Gain), SMARCA4 (3.0, Gain), SMARCB1 (1.1,Loss), STK11 (3.0,Gain), TP53 (3.3,Gain), and ERBB2 (3.3,Gain).

The mother’s family had a history of a high incidence of tumors (father, lung cancer; mother, cervical cancer; grandfather, liver cancer; grandmother, lung cancer; her father had 5 siblings, among whom her father’s brother had lung cancer, with no tumors in the others) (**Supplementary Figure 3**). Given that Patient one’s brother also had MB and with the family history of tumors, Patient one and his parents were tested for gene mutations after consent of the family members. Important genes related to solid tumors in children were examined using next-generation sequencing, with the detection range covering 998 genes and mainly including base substitution (single-nucleotide variant, SNV), small insertion/deletion (indel), copy number variation (CNV), gene fusion, microsatellite instability (MSI), tumor mutational burden (TMB) and tumor genetic susceptibility gene analysis. In Patient one, testing found a germline mutation in the SUFU gene (NM_016169:exon3:c.C436T:p.R146X), with a mutation frequency of 97.3% (**Supplementary Table 1**). The child’s mother also carried the SUFU germline mutation, whereas the father was negative. Other tumor-related gene variants include NF1 (NM_001042492:exon16:c.C1765T:p.Q589X), BRCA1 (gain), ERBB2 (gain), SMARCA4 (gain), SMARCB1 (loss), STK11 (gain), and TP53 (gain) (see **Figure 2** for CNV variation information). The distribution of mutations in Patient one is illustrated by a Circos diagram in **Supplementary Figure 4**. Mutation in the SUFU gene can cause Gorlin-Goltz syndrome, which is closely related to MB. Based on the medical history of Patient two, genetic testing was also performed, and the SUFU gene mutation was detected. According to the diagnostic criteria of Gorlin-Goltz syndrome, Patients one and two and their mother were diagnosed with the syndrome (**Table 1**). Comprehensive analysis of the pathological examination and gene mutation detection results led to a diagnosis of MBEN (MB with extensive nodularity) as the histological type for Patient one; the molecular type was SHH-activated MB with wild-type TP53.

**Table 1:** Criteria for Gorlin-Goltz syndrome.

			Criteria	Case 1	Case 2
**Major**		1	Multiple (>2) basal cell carcinomas or one diagnosed before 20 years of age	No	No
		2	Keratocystic odontogenic tumor before 20 years of age	No	No
		3	Palmar or plantar pitting	No	No
		4	Lamellar calcification of the falx cerebri	No	No
		5	Medulloblastoma, typically desmoplastic	**Yes**	**Yes**
		6	Family history of NBCCS(First degree relative)	**Yes**	**Yes**
**Minor**		1	Rib abnormalities	No	No
		2	Other specific skeletal malformations and radiologic changes	No	No
		3	Macrocephaly	No	No
		4	Cleft lip or palate	No	No
		5	Ovarian or cardiac fibroma	No	No
		6	Lymphomesenteric cysts	No	No
		7	Ocular abnormalities	No	No
**Molecular confirmation**		1	SUFU mutation	**Yes**	**Yes**
		2	PTCH1 mutation	No	No
	3		PTCH2 mutation	No	No

Diagnostic criteria of NBCCS (1): two major criteria (2); one major and two minor criteria (3); one major criterion with molecular confirmation.

One month after the operation, Patient one received chemotherapy in the Oncology Department (scheme: HEAD START4: VCR 0.05 mg/kg d1,d8+CDDP 35 mg/m2 d1+VP16 4mg/kg d2,d3+CTX 65 mg/kg d2,d3+HDMTX 400 mg/kg d4); the chemotherapy regimen was repeated every three weeks. After chemotherapy was complete, Patient one underwent umbilical cord blood stem cell transplantation(UCBT) (from his mother) at 4 months after surgery. The treatment of Patient one in detail was showed in **Supplementary Figure 5**. Follow-up at 10 months showed no recurrence of the tumor by MRI, and the child was in good condition.

## Discussion

Gorlin-Goltz syndrome, also known as nevoid basal cell carcinoma syndrome (NBCCS), is a rare autosomal dominant disorder with the primary manifestations of congenital dysplasia and susceptibility to certain tumors (6). Developmental abnormalities mainly include overgrowth, developmental delay, bone abnormalities and polydactyly, intracranial calcification, and small pink depressions on the hands and feet. Related tumors include keratocystic odontogenic tumor (KCOT), basal cell carcinoma, medulloblastoma, and meningioma, among others. Multiple KCOTs can occur in the jaw, which is one of the most common manifestations of this syndrome. The 2011 revised Gorlin-Goltz syndrome diagnostic criteria include 6 major criteria and 6 minor criteria. Gorlin-Goltz syndrome is diagnosed when patients meet two major criteria, one major criterion and two minor criteria, or one major criterion with a positive genetic test result (7). The 2 children and their mother reported in this paper can be diagnosed with Gorlin-Goltz syndrome according to these criteria. The diagnosis in this study was mainly based on SUFU gene mutation, family history, MB presence, and odontogenic keratocyst s(their mother); interestingly, neither of the patients in this study had other related disorders of Gorlin-Goltz syndrome. Therefore, due to the lack of other symptoms to assist in diagnosis, diagnosis is difficult for infants and young children, and genetic testing is often an important measure for early diagnosis.

Gorlin-Goltz syndrome is a hereditary syndrome, and mutated genes include PTCH1, SUFU, and PTCH2, among others. PTCH1 mutation is relatively common, accounting for 60% to 75%, but the probability of MB after SUFU mutation is 20 times that of PTCH1 mutation (8, 9). SUFU gene germline mutations are mainly seen in infant SHH-subtype MB and are closely related to infantile desmoplastic/extensive nodular MB, of which 21% of cases involve SUFU germline mutations (10). Approximately 20% of MBs with SUFU germline mutations involve Gorlin-Goltz syndrome, and the age of onset is under 3 years of age (11). Reports of MB in infants less than 6 months of age with Gorlin-Goltz syndrome caused by SUFU gene mutation are very rare. This article reviews relevant case reports in the past 10 years(A Medline search from January 2010 to December 2021 using the key phrase “medulloblastoma” and “SUFU”). Only 10 cases less than 6 months of age have been reported (6 males and 4 females), and 80% of the histological types were MB with extensive nodularity; there were 3 cases of recurrence and 4 deaths. MBEN constitutes 3% of medulloblastomas, and occurs in children less than 3 years of age. Numerous patients with MBEN are treated by tumour resection and chemotherapy, without radiotherapy, and have a favourable outcome, which is probably related to spontaneous neurocytic (as well as astrocytic) differentiation of this type of medulloblastoma (12, 13).The relevant mutation information and prognostic data are shown in **Table 2**.

**Table 2:** Summary of the past reported case series of germline SUFU-mutated medulloblastoma in patients younger than 6 months of age.

Author (date)	Country	Sex, Age(m)	HS	MD	SUFUMutation	NBCCS	Inheritance	FHM	Treatment	Outcome
Gershanov S (2021) (14)	Israel	Female,3	MBEN	NA	*De novo* heterozygous loss of exon 3	NA	No	No	PR+No chemotherapy	Follow-up 6 years, no recurrence, alive
Andrey Korshunov(2018) (15)	Germany	Male,5	MBEN	SHH	SUFU(p.260 fs)	No	NA	NA	GTR+ IV MTX	Follow-up 42 months, recurrence, alive
		Male,5 mon	MBEN	SHH	SUFU(p.W136X)	No	NA	NA	GTR+ IV MTX	Follow-up 34 months, recurrence, alive
Guerrini-Rousseau L (2018) (11)	France	Male,1 (siblings)	MBEN	NA	c.71del p.Pro24Argfs72	No	Inherited from mother	NA	Biopsy+ CC	Follow-up 0.04 years,died
		Male, 3 (siblings)	MBEN	NA	c.71del p.Pro24Argfs72	No	Inherited from mother	NA	Only PR	Follow-up 0.03 years,died
		Female, 6	DNMB	NA	c.1096_1117delinsGAA p.Leu366Glufs14	No	Inherited from father	NA	CR+ CC	Follow-up 2.4 years, no recurrence,alive
		Male, 1	MBEN	NA	c.1022+1G>A	No	NA	NA	PR+ CC	Follow-up 1.4 years, Died
Brugières L(2010) (16)	France	Male, < 3	MBEN	NA	c.71del	NA	Inherited from mother	YES	NA	Died
		Female, < 1	MBEN	NA	c.71dup	NA	Inherited	YES	NA	Alive after relapse
		Female, < 6	DNMB	NA	c.71dup	NA	Inherited	YES	CR	Alive in complete remission

HS, histological subtype; MD, molecular diagnosis; NBCCS, nevoid basal cell carcinoma syndrome; FHM, family history of malignancy; PR, partial resection; CR, complete resection; NA, not available; GTR, gross total resection; IV MTX, intravenous methotrexate injection; CC, conventional chemotherapy; MBEN, medulloblastoma with extensive nodularity; DNMB, desmoplastic/nodular medulloblastoma.

This sample detected SUFU gene c.C436T (p.R146X). No mutations were detected in PTCH1 or PTCH2. It has been reported that in a mouse model, a homozygous variant of SUFU causes protein inactivation and cannot inhibit transcriptional activation, with GLI3 not expressed (17). This variant was found in only one literature report of a patient with MB (18). SUFU, a tumor-suppressor gene, encodes a fusion protein suppressor that is part of the hedgehog signaling pathway and one of the key regulators of embryonic development. SUFU sequesters GLI transcription factors in the cytoplasm and inhibit their activity (19). SUFU inhibits Wnt signaling by exporting β-catenin from the nucleus, and it also acts as an interaction point between the hedgehog and p63 signaling pathways, which may be an important aspect of keratinocyte differentiation regulation (20). Germline or somatic mutations of the truncated type of SUFU, often accompanied by loss of the wild-type allele, are closely associated with childhood MB (21). Germline mutations in SUFU are associated with nevoid basal cell carcinoma syndrome, homozygous mutations with Joubert syndrome type 32, and heterozygous germline mutations with susceptibility to familial meningiomas (9, 22–24). In addition, this study detected an NF1 somatic mutation (NM_001042492:exon16:c.C1765T:p.Q589X). Germline mutations in NF1 have been reported in predisposition syndrome neurofibromatosis type 1, along with somatic mutations in many tumor types, including breast cancer and melanoma (25). The NF1 Q589X mutation detected in this sample is a nonsense mutation. Many gene CNVs were also detected in the patient, such as BRCA1 gain, ERBB2 gain, SMARCA4 gain, SMARCB1 loss, STK11 gain, and TP53 gain. However, there is no literature suggesting that TP53 gain, BRCA1 gain, SMARCA4 gain, and STK11 gain are related to MB. Although TP53 gain was found in this study, no mutation was detected. When TP53 is amplified, detection of TP53 gene mutation by immunohistochemistry may be positive, and it is easy to diagnose the TP53 mutant type, which needs attention. SMARCB1 gene mutation is extremely rare in MB. SMARCB1 gene mutation was detected in 1 case of MB, but its clinical significance remains unclear (26).

The ERBB2 gene, also known as human epidermal growth factor receptor 2 (HER2), encodes a transmembrane receptor that belongs to the ERBB family of receptor tyrosine kinases. Activation of ERBB2-mediated signals can ultimately initiate cell proliferation, migration and differentiation (27). Studies have found that ERBB2 is a therapeutic target for a variety of adult or pediatric tumors, including MB, and ERBB2 gene mutation occurs in 40% of MBs. MB patients with ERBB2 amplification have worse overall and progression-free survival, and this type may be more aggressive (28). Clinical studies of ERBB2-targeted drugs for treatment of ERBB2-amplified MB are currently underway. The results of a phase 1 interim analysis have demonstrated the feasibility of ERBB2-specific CAR-T cells for treatment of children and young patients with relapsed/refractory CNS tumors, including MB (29).

In general, Gorlin-Goltz syndrome screening has important clinical significance for the diagnosis and treatment of MB. Genetic counselling and testing should be used as a standard-of-care procedure in patients with MBWNT and MBSHH, because these patients have the highest prevalence of damaging germline mutations in known cancer predisposition genes (10). Chemotherapy strategies for young children with MB have been explored, the pilot trial HIT-SKK’87 confirmed that postoperative chemotherapy may successfully delay the start of radiotherapy for children aged < 3 years; In the subsequent HIT-SKK’92 trial, intraventricular methotrexate was introduced as a substitute for radiotherapy, and favorable survival rates have been obtained, especially for young patients with desmoplastic/nodular MB, and neurocognitive deficits were less pronounced compared with the HIT-SKK’87 trial (30). In 2011, the trial HIT-SKK’2000BIS4 confirmed that sustained tumor control can be achieved in young children with desmoplastic/nodular MB variants (13). Furthermore, studies also reported encouraging survival, quality of life (QoL) and neurocognitive outcome data for medulloblastoma patients treated on “Head Start” (HS) I and II trial (31). In 2020, “Head Start” III, a clinical trial using intensive induction followed by myeloablative chemotherapy and autologous hematopoietic cell rescue (AuHCR), showed excellent survival and preservation of mean IQ and memory for young children with Nodular/desmoplastic medulloblastoma (32). But in comparison, the adequate strategy for young children with nodular/desmoplastic MB or MB with extensive nodularity might be HIT SKK chemotherapy or similar approaches at present. Chemotherapy is the first-line treatment for MB patients with Gorlin-Goltz syndrome; radiotherapy is not recommended and should be avoided as much as possible because the incidence of secondary tumors (basal cell carcinoma, meningioma, etc.) increases significantly after radiotherapy. Therefore, radiological examinations, such as X-ray and CT, should also be reduced as much as possible in such patients. SMO inhibitors (e.g., sonidegib, vismodegib) have also been used as treatment for SHH-subtype MB. It has been reported that the progression-free survival time is significantly prolonged in SHH-subtype patients after the application of SMO inhibitors, and compared with PTCH1 mutation, targeted therapy can achieve better results in patients with SUFU mutation (33). In recent years, there has been great progress in the treatment of MB with hematopoietic stem cell transplantation. Infusing hematopoietic stem cells after radiotherapy/chemotherapy can rebuild the recipient’s hematopoietic and immune system to treat disease. In this study, Patient two visited a local hospital(not our hospital),which is an ordinary hospital and not able to carry out operations for children with intracranial tumor, in addition, the child’s parents did not know enough about the disease at that time, and they mistakenly believed that the prognosis of the disease is not good (actually the prognosis is good), they gave up all treatments. We thought that Patient two was not treated enough. Patient one had the same tumor(at the same location), the parents were desperate and decided to save this child at all costs, and would try some emerging treatments, including stem cell therapy and chemotherapy regimens that were in clinical trial stage. Fortunately, umbilical cord blood of his mother was stored during delivery. After thorough discussions with parents, HEAD START4 regimen combined with umbilical cord blood stem cell transplantation were selected for Patient one (based on a prospective randomized clinical trial, gov Identifier: NCT02875314). Maybe, Patient one was “overtreated”, however, fortunately, the patient recovered very well at present. Till now, the child’s condition is very good. There is no tumor recurrence on MRI. Growth and development are similar to those of his peers. It has been reported that for high-risk and recurrent MB, reduced-dose cranial radiotherapy combined with high-dose chemotherapy and autologous stem cell transplantation can significantly reduce the complications caused by myelosuppression and improve the survival rate of children (34). Therefore, high-dose chemotherapy combined with stem cell transplantation is expected to be the first-line treatment for high-risk and recurrent MB (35).

## Data availability statement

The datasets presented in this study can be found in online repositories. The names of the repository/repositories and accession number(s) can be found in the article/**Supplementary Material**.

## Ethics statement

This study was reviewed and approved by Institutional Review Board of Xinhua Hospital Affiliated to Shanghai Jiao Tong University School of Medicine. Written informed consent to participate in this study was provided by the participants’ legal guardian/next of kin.

## Author contributions

YC contributed to material preparation and data analysis. JM and YZ conceived the idea and directed the whole project. HZ contributed to review and technical assistance. YC wrote the paper, and all authors were involved in editing the manuscript. All authors contributed to the article and approved the submitted version.

## Funding

This study received funding from the Shanghai Xin Hua Hospital (JZPI201701 to JM).

## Acknowledgments

We appreciate the patients’ approval for sharing their stories and all research staff’s efforts in this report.

## Conflict of interest

The authors declare that the research was conducted in the absence of any commercial or financial relationships that could be construed as a potential conflict of interest.

## Publisher’s note

All claims expressed in this article are solely those of the authors and do not necessarily represent those of their affiliated organizations, or those of the publisher, the editors and the reviewers. Any product that may be evaluated in this article, or claim that may be made by its manufacturer, is not guaranteed or endorsed by the publisher.

## Supplementary material

Supplementary Figure 1 MRI findings for Patient two. MRI showed a large pathological mass in the posterior fossa, very similar to the imaging for Patient 1. **(A)** Axial T1 images. **(B)** Sagittal T1 images.

Supplementary Figure 2 Postoperative review and pathological results for Patient one. The tumor was gray–white, fish-like, and had an abundant blood supply **(A)**. Postoperative CT and MRI showed complete tumor resection **(B–D)**. HE staining **(E, F)**.

Supplementary Figure 3 Pedigree of the family.

Supplementary Figure 4 Distribution of mutations in Patient one depicted by a Circos diagram.

Supplementary Figure 5 The treatment of Patient one in detail.

## References

[1] Lawson C Ahmed Alta TB Moschou G Skamnaki V Solovou TGA Topham C . Novel diarylamides and diarylureas with n-substitution dependent activity against medulloblastoma. Eur J Med Chem (2021) 225:113751. doi: 10.1016/j.ejmech.2021.113751 34391032

[2] Cornejo P Feygin T Vaughn J Pfeifer CM Korostyshevska A Patel M . Imaging of fetal brain tumors. Pediatr Radiol (2020) 50(13):1959–73. doi: 10.1007/s00247-020-04777-z 33252762

[3] Woodward PJ Sohaey R Kennedy A Koeller KK . From the archives of the AFIP: a comprehensive review of fetal tumors with pathologic correlation. Radiographics (2005) 25(1):215–42. doi: 10.1148/rg.251045156 15653597

[4] Korostyshevskaya AM Savelov AA Papusha LI Druy AE Yarnykh VL . Congenital medulloblastoma: Fetal and postnatal longitudinal observation with quantitative MRI. Clin Imaging. (2018) 52:172–6. doi: 10.1016/j.clinimag.2018.06.001 30096555

[5] Verkouteren BJA Cosgun B Vermeulen RJ Reinders M van Geel M Gille JJP . Prevalence of medulloblastoma in basal cell nevus syndrome patients with a PTCH1 mutation. Neuro Oncol (2021) 23(6):1035–6. doi: 10.1093/neuonc/noab048 PMC8168812 33864364

[6] Gorlin RJ . Nevoid basal cell carcinoma (Gorlin) syndrome. Genet Med (2004) 6(6):530–9. doi: 10.1097/01.GIM.0000144188.15902.C4 15545751

[7] Bree AF Shah MR Group BC . Consensus statement from the first international colloquium on basal cell nevus syndrome (BCNS). Am J Med Genet A (2011) 155A(9):2091–7. doi: 10.1002/ajmg.a.34128 21834049

[8] Evans DG Oudit D Smith MJ Rutkowski D Allan E Newman WG . First evidence of genotype-phenotype correlations in gorlin syndrome. J Med Genet (2017) 54(8):530–6. doi: 10.1136/jmedgenet-2017-104669 28596197

[9] Smith MJ Beetz C Williams SG Bhaskar SS O'Sullivan J Anderson B . Germline mutations in SUFU cause gorlin syndrome-associated childhood medulloblastoma and redefine the risk associated with PTCH1 mutations. J Clin Oncol (2014) 32(36):4155–61. doi: 10.1200/JCO.2014.58.2569 25403219

[10] Waszak SM Northcott PA Buchhalter I Robinson GW Sutter C Groebner S . Spectrum and prevalence of genetic predisposition in medulloblastoma: a retrospective genetic study and prospective validation in a clinical trial cohort. Lancet Oncol (2018) 19(6):785–98. doi: 10.1016/S1470-2045(18)30242-0 PMC5984248 29753700

[11] Guerrini-Rousseau L Dufour C Varlet P Masliah-Planchon J Bourdeaut F Guillaud-Bataille M . Germline SUFU mutation carriers and medulloblastoma: clinical characteristics, cancer risk, and prognosis. Neuro Oncol (2018) 20(8):1122–32. doi: 10.1093/neuonc/nox228 PMC6280147 29186568

[12] Giangaspero F Perilongo G Fondelli MP Brisigotti M Carollo C Burnelli R . Medulloblastoma with extensive nodularity: a variant with favorable prognosis. J Neurosurg (1999) 91(6):971–7. doi: 10.3171/jns.1999.91.6.0971 10584843

[13] von Bueren AO von Hoff K Pietsch T Gerber NU Warmuth-Metz M Deinlein F . Treatment of young children with localized medulloblastoma by chemotherapy alone: results of the prospective, multicenter trial HIT 2000 confirming the prognostic impact of histology. Neuro Oncol (2011) 13(6):669–79. doi: 10.1093/neuonc/nor025 PMC3107096 21636711

[14] Gershanov S Toledano H Pernicone N Fichman S Michowiz S Pinhasov A . Differences in RNA and microRNA expression between PTCH1- and SUFU-mutated medulloblastoma. Cancer Genomics Proteomics. (2021) 18(3):335–47. doi: 10.21873/cgp.20264 PMC8126327 33893086

[15] Korshunov A Sahm F Stichel D Schrimpf D Ryzhova M Zheludkova O . Molecular characterization of medulloblastomas with extensive nodularity (MBEN). Acta Neuropathol. (2018) 136(2):303–13. doi: 10.1007/s00401-018-1840-0 29569031

[16] Brugieres L Pierron G Chompret A Paillerets BB Di Rocco F Varlet P . Incomplete penetrance of the predisposition to medulloblastoma associated with germ-line SUFU mutations. J Med Genet (2010) 47(2):142–4. doi: 10.1136/jmg.2009.067751 19833601

[17] Makino S Zhulyn O Mo R Puviindran V Zhang X Murata T . T396I mutation of mouse sufu reduces the stability and activity of Gli3 repressor. PloS One (2015) 10(3):e0119455. doi: 10.1371/journal.pone.0119455 25760946 PMC4356511

[18] Al-Rahawan MG Trevino S Jacob R Murray JC Al-Rahawan MM . Medulloblastoma in a toddler with gorlin syndrome. Proc (Bayl Univ Med Cent). (2018) 31(2):216–8. doi: 10.1080/08998280.2018.1435111 PMC5914436 29706825

[19] Kogerman P Grimm T Kogerman L Krause D Unden AB Sandstedt B . Mammalian suppressor-of-fused modulates nuclear-cytoplasmic shuttling of gli-1. Nat Cell Biol (1999) 1(5):312–9. doi: 10.1038/13031 10559945

[20] Meng X Poon R Zhang X Cheah A Ding Q Hui CC . Suppressor of fused negatively regulates beta-catenin signaling. J Biol Chem (2001) 276(43):40113–9. doi: 10.1074/jbc.M105317200 11477086

[21] Kool M Jones DT Jager N Northcott PA Pugh TJ Hovestadt V . Genome sequencing of SHH medulloblastoma predicts genotype-related response to smoothened inhibition. Cancer Cell (2014) 25(3):393–405. doi: 10.1016/j.ccr.2014.02.004 24651015 PMC4493053

[22] Aavikko M Li SP Saarinen S Alhopuro P Kaasinen E Morgunova E . Loss of SUFU function in familial multiple meningioma. Am J Hum Genet (2012) 91(3):520–6. doi: 10.1016/j.ajhg.2012.07.015 PMC3511996 22958902

[23] De Mori R Romani M D'Arrigo S Zaki MS Lorefice E Tardivo S . Hypomorphic recessive variants in SUFU impair the sonic hedgehog pathway and cause joubert syndrome with cranio-facial and skeletal defects. Am J Hum Genet (2017) 101(4):552–63. doi: 10.1016/j.ajhg.2017.08.017 PMC5630196 28965847

[24] Pastorino L Ghiorzo P Nasti S Battistuzzi L Cusano R Marzocchi C . Identification of a SUFU germline mutation in a family with gorlin syndrome. Am J Med Genet A (2009) 149A(7):1539–43. doi: 10.1002/ajmg.a.32944 19533801

[25] Hodis E Watson IR Kryukov GV Arold ST Imielinski M Theurillat JP . A landscape of driver mutations in melanoma. Cell (2012) 150(2):251–63. doi: 10.1016/j.cell.2012.06.024 PMC3600117 22817889

[26] El-Ayadi M Egervari K Merkler D McKee TA Gumy-Pause F Stichel D . Concurrent IDH1 and SMARCB1 mutations in pediatric medulloblastoma: A case report. Front Neurol (2018) 9:398. doi: 10.3389/fneur.2018.00398 29971034 PMC6018091

[27] Yarden Y Shilo BZ . SnapShot: EGFR signaling pathway. Cell (2007) 131(5):1018. doi: 10.1016/j.cell.2007.11.013 18045542

[28] Nellan A Rota C Majzner R Lester-McCully CM Griesinger AM Mulcahy Levy JM . Durable regression of medulloblastoma after regional and intravenous delivery of anti-HER2 chimeric antigen receptor T cells. J Immunother Cancer. (2018) 6(1):30. doi: 10.1186/s40425-018-0340-z 29712574 PMC5925833

[29] Vitanza NA Johnson AJ Wilson AL Brown C Yokoyama JK Kunkele A . Locoregional infusion of HER2-specific CAR T cells in children and young adults with recurrent or refractory CNS tumors: an interim analysis. Nat Med (2021) 27(9):1544–52. doi: 10.1038/s41591-021-01404-8 34253928

[30] Timmermann B Kortmann RD Kuhl J Rutkowski S Dieckmann K Meisner C . Role of radiotherapy in anaplastic ependymoma in children under age of 3 years: results of the prospective German brain tumor trials HIT-SKK 87 and 92. Radiother Oncol (2005) 77(3):278–85. doi: 10.1016/j.radonc.2005.10.016 16300848

[31] Dhall G Grodman H Ji L Sands S Gardner S Dunkel IJ . Outcome of children less than three years old at diagnosis with non-metastatic medulloblastoma treated with chemotherapy on the "Head start" I and II protocols. Pediatr Blood Cancer. (2008) 50(6):1169–75. doi: 10.1002/pbc.21525 18293379

[32] Dhall G O'Neil SH Ji L Haley K Whitaker AM Nelson MD . Excellent outcome of young children with nodular desmoplastic medulloblastoma treated on "Head start" III: a multi-institutional, prospective clinical trial. Neuro Oncol (2020) 22(12):1862–72. doi: 10.1093/neuonc/noaa102 PMC7746930 32304218

[33] Robinson GW Orr BA Wu G Gururangan S Lin T Qaddoumi I . Vismodegib exerts targeted efficacy against recurrent sonic hedgehog-subgroup medulloblastoma: Results from phase II pediatric brain tumor consortium studies PBTC-025B and PBTC-032. J Clin Oncol (2015) 33(24):2646–54. doi: 10.1200/JCO.2014.60.1591 PMC4534527 26169613

[34] Sung KW Lim DH Son MH Lee SH Yoo KH Koo HH . Reduced-dose craniospinal radiotherapy followed by tandem high-dose chemotherapy and autologous stem cell transplantation in patients with high-risk medulloblastoma. Neuro Oncol (2013) 15(3):352–9. doi: 10.1093/neuonc/nos304 PMC3578484 23258845

[35] Aihara Y Tsuruta T Kawamata T Kanno H Maebayashi K Sakauchi M . Double high-dose chemotherapy followed by autologous peripheral blood stem cell transplantation for primary disseminated medulloblastoma: a report of 3 cases. J Pediatr Hematol Oncol (2010) 32(2):e70–4. doi: 10.1097/MPH.0b013e3181c46b92 20168248

