# Supplementary figures and images for: Congenital medulloblastoma in two brothers with SUFU-mutated Gorlin-Goltz syndrome: Case reports and literature review
Source: Front Oncol. 2022 Oct 12;12:988798. doi: 10.3389/fonc.2022.988798 (PMC9603755; doi:10.3389/fonc.2022.988798)

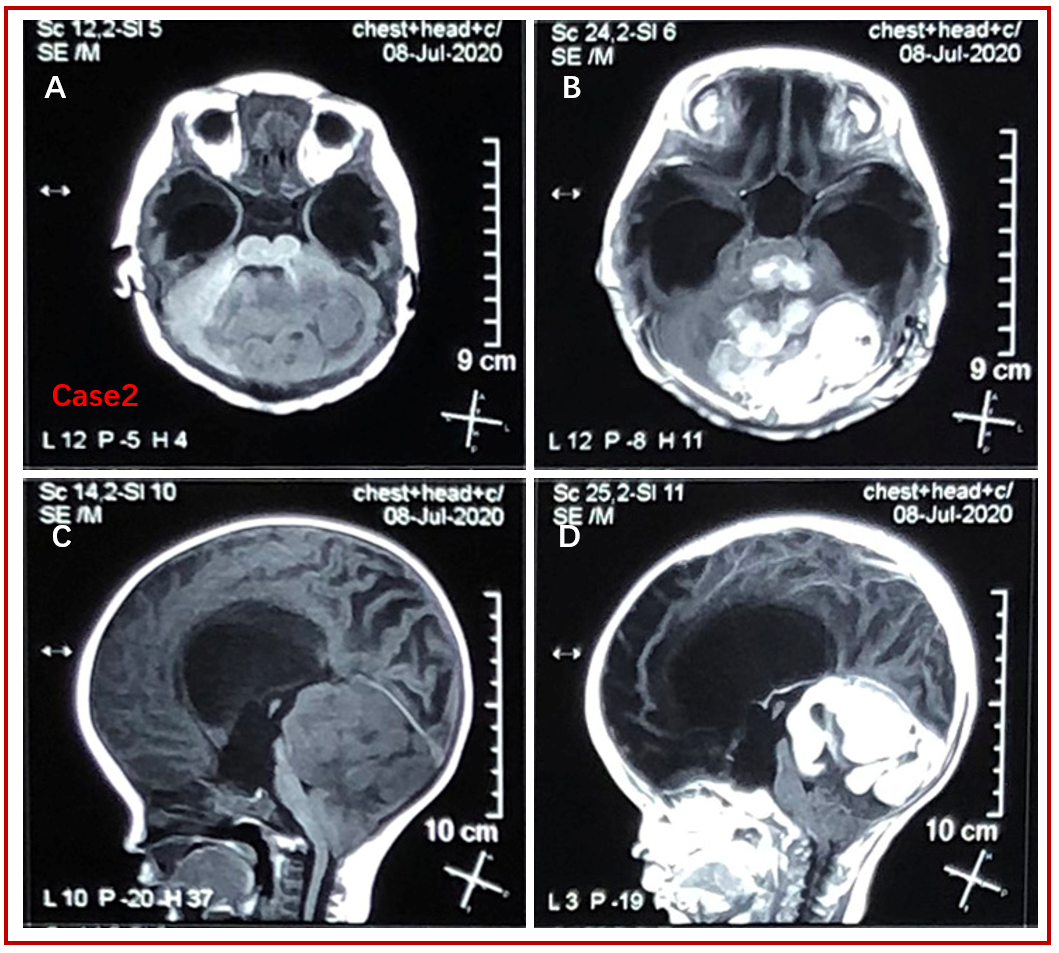

Supplement: Supplementary Figure 1 — MRI findings for Patient two. MRI showed a large pathological mass in the posterior fossa, very similar to the imaging for Patient 1. (A) Axial T1 images. (B) Sagittal T1 images. [file Image_1.tif]

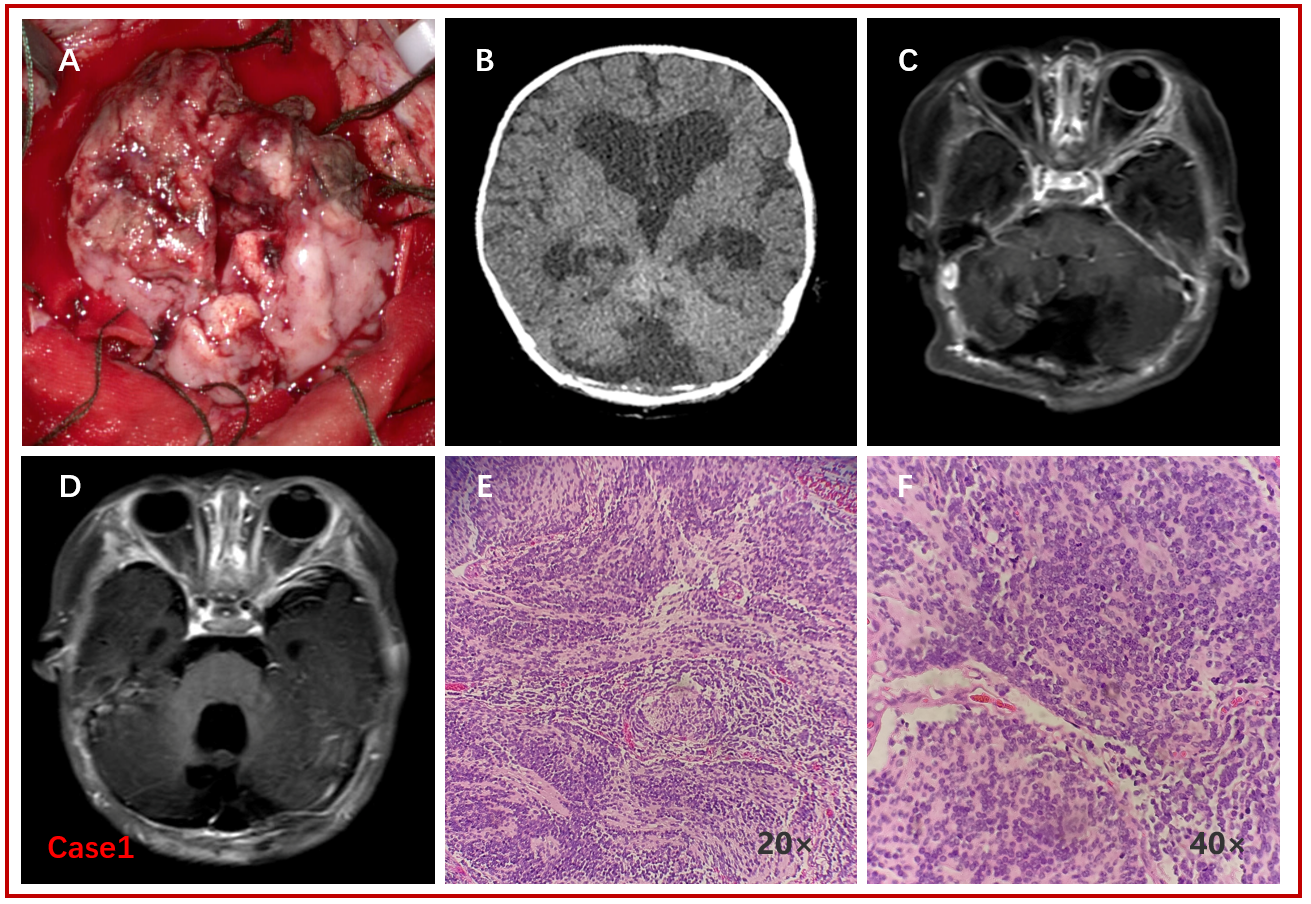

Supplement: Supplementary Figure 2 — Postoperative review and pathological results for Patient one. The tumor was gray–white, fish-like, and had an abundant blood supply (A). Postoperative CT and MRI showed complete tumor resection (B–D). HE staining (E, F). [file Image_2.tif]

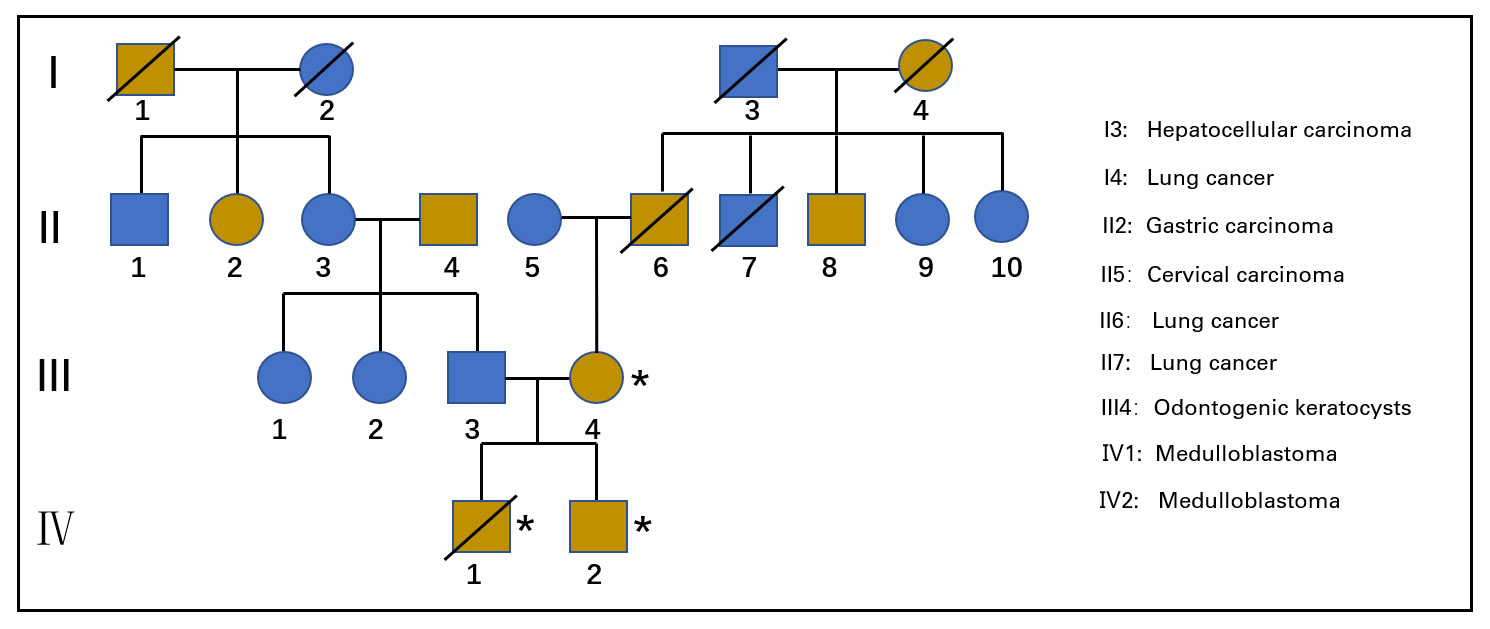

Supplement: Supplementary Figure 3 — Pedigree of the family. [file Image_3.tif]

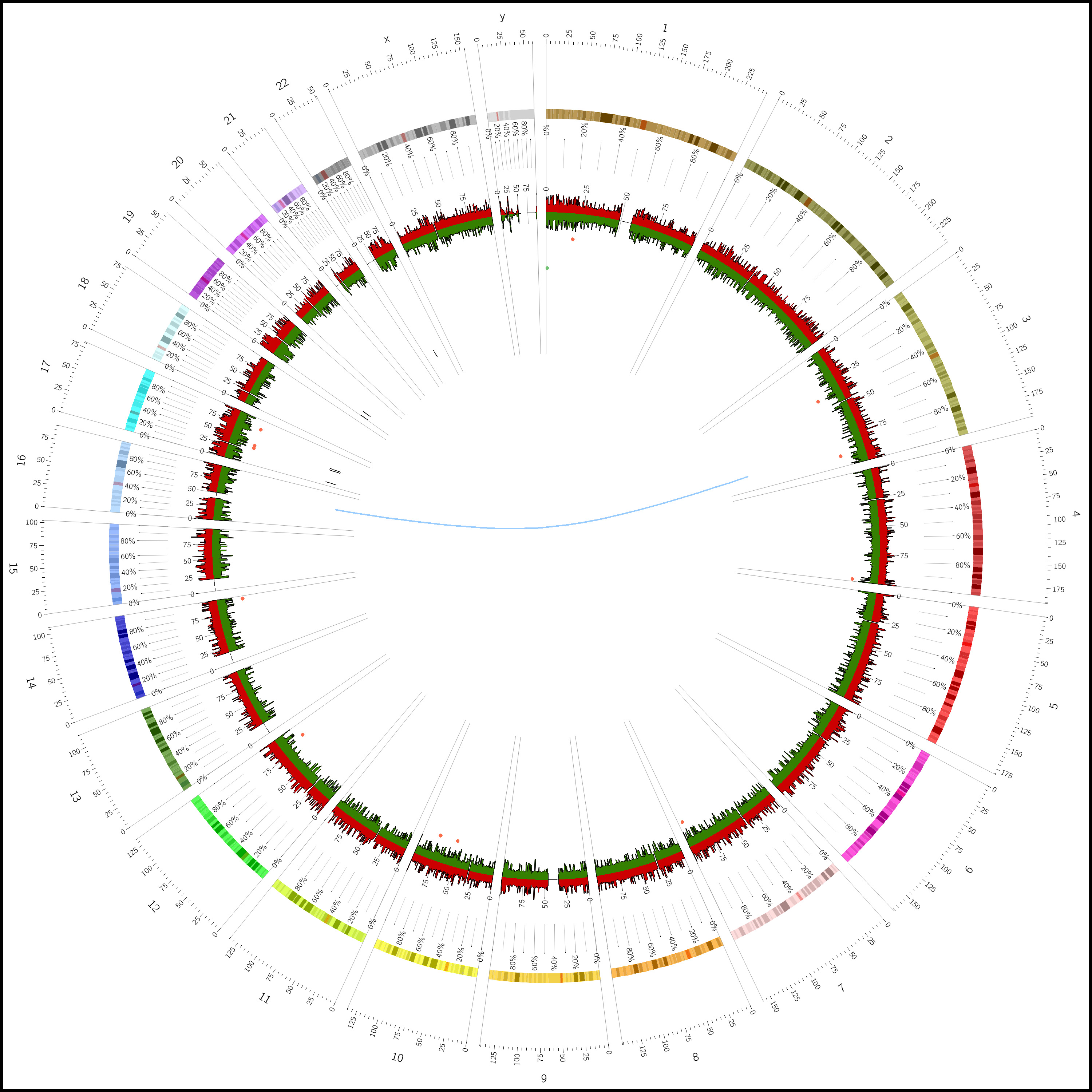

Supplement: Supplementary Figure 4 — Distribution of mutations in Patient one depicted by a Circos diagram. [file Image_4.tif]

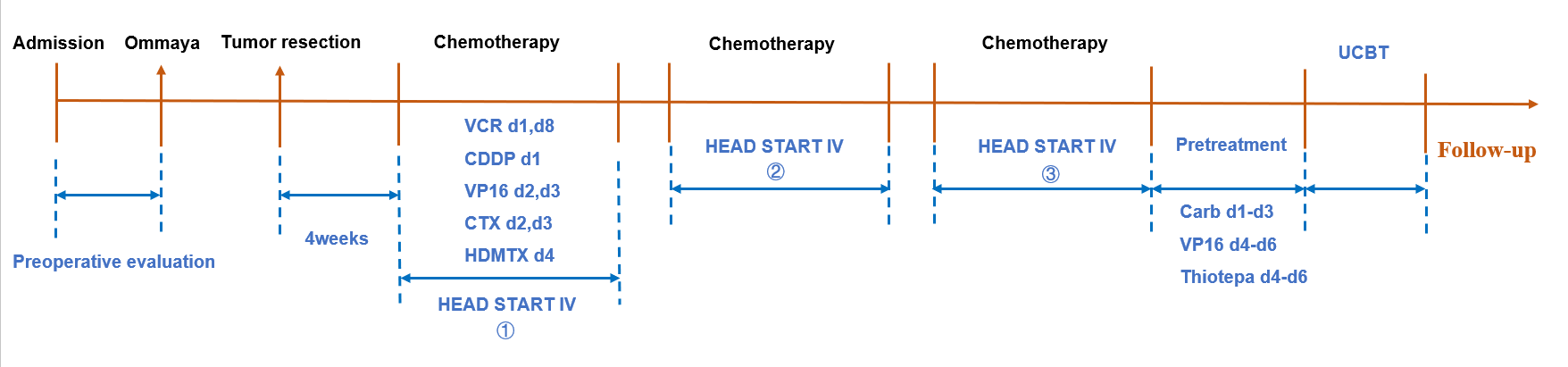

Supplement: Supplementary Figure 5 — The treatment of Patient one in detail. [file Image_5.tif]
