# Supplementary material for: Congenital medulloblastoma in two brothers with SUFU-mutated Gorlin-Goltz syndrome: Case reports and literature review
Source: Front Oncol. 2022 Oct 12;12:988798. doi: 10.3389/fonc.2022.988798 (PMC9603755; doi:10.3389/fonc.2022.988798)
Supplement: Supplementary file 6 [file Table_1.docx]

**Supplementary Table1. Genetic mutation analysis for Patient one.**

| **Gene** | **Genetic variation** | **Chromosomal Location** | **Frequency** | **Sequencing depth（×）** | **PopFreq**  **Max^a^** | **SIFT^b^** | **Type** | **Classification of pathogenicity^c^** |
| --- | --- | --- | --- | --- | --- | --- | --- | --- |
| **SUFU** | NM_016169:exon3:c.C436T:p.R146X | chr10:104309  845C>T | 97.30% | 2141 | 6.46E-05 | . | G | P |
| **NF1** | NM_001042492:exon16:c.C1765T:p.Q589X | chr17:295505  05C>T | 1.80% | 709 | 0 | . | S | Grade2 |
| **TNFRSF14** | NM_003820:exon8:c.843dupA:p.N282Kfs*58 | chr1:2494702  ->A | 50.30% | 3082 | 0 | . | S | Grade 3 |
| **TAL1** | NM_003189:exon6:c.T962C:p.M321T | chr1:4768542  6A>G | 50.70% | 5128 | 0 | 0.045 | S | Grade 3 |
| **PRF1** | NM_005041:exon3:c.C673T:p.R225W | chr10:723588  04G>A | 2.90% | 5304 | 0.0001 | 0.025 | S | Grade 3 |
| **PTPN11** | NM_002834:exon9:c.C1010T:p.T337M | chr12:112915  737C>T | 1.30% | 1121 | 0 | 0 | S | Grade 3 |
| **HSP90AA1** | NM_001017963:exon12:c.C2545T:p.R849C | chr14:102548  069G>A | 1.50% | 503 | 0 | 0 | S | Grade 3 |
| **NF1** | NM_001042492:exon16:c.G1802A:p.R601Q | chr17:295505  42G>A | 2.00% | 699 | 0 | 0.005 | S | Grade 3 |
| **NF1** | NM_001042492:exon21:c.T2473C:p.S825P | chr17:295561  06T>C | 3.50% | 1054 | 0 | 0.051 | S | Grade 3 |
| **NF1** | NM_001042492:exon21:c.A2507C:p.E836A | chr17:295561  40A>C | 1.40% | 1116 | 0 | 0.008 | S | Grade 3 |
| **NF1** | NM_001042492:exon32:c.A4307G:p.E1436G | chr17:295854  95A>G | 2.70% | 540 | 0 | 0.017 | S | Grade 3 |
| **SLFN11** | NM_001104589:exon6:c.C2150T:p.P717L | chr17:336799  31G>A | 47.10% | 784 | 0.0003 | 0.38 | S | Grade 3 |
| **PRKCA** | NM_002737:exon5:c.C527G:p.T176R | chr17:646416  27C>G | 48.80% | 2519 | 0 | 0.357 | S | Grade 3 |
| **PIK3CA** | NM_006218:exon2:c.T31G:p.W11G | chr3:1789166  44T>G | 1.60% | 593 | 0 | 0.021 | S | Grade 3 |
| **PPP4R2** | NM_174907:exon3:c.A197C:p.E66A | chr3:7309641  7A>C | 5.00% | 602 | 0 | 0.063 | S | Grade 3 |
| **FAT1** | NM_005245:exon10:c.A6830G:p.N2277S | chr4:1875409  10T>C | 48.70% | 2786 | 0 | 0 | S | Grade 3 |
| **KMT2C** | NM_170606:exon19:c.G3143A:p.G1048D | chr7:1519215  35C>T | 1.40% | 925 | 0 | 0.021 | S | Grade 3 |
| **KMT2C** | NM_170606:exon14:c.G2020A:p.E674K | chr7:1519454  99C>T | 1.30% | 683 | 0 | 0.61 | S | Grade 3 |

a: PopFreqMax represents the maximum allele frequency in the population carrying the mutation. b: SIFT predicts whether a mutation is harmful; greater than 0.05 is T (tolerance), and less than or equal to 0.05 is D (deleterious). c: Somatic mutation evaluation is divided into 3 categories (grade 1: variants clearly associated with disease; grade 2: variants likely to be associated with disease; grade 3: variants of unknown clinical significance), and germline mutation is divided into 5 categories (pathogenic, P; likely pathogenic, LP; uncertain

significance, VUS; likely benign,LB, benign; B, germline mutation; S, somatic mutation).
